# Supplementary material for: Qualitative process evaluation from a complex systems perspective: A systematic review and framework for public health evaluators
Source: PLoS Med. 2020 Nov 2;17(11):e1003368. doi: 10.1371/journal.pmed.1003368 (PMC7605618; doi:10.1371/journal.pmed.1003368)
Supplement: S1 Text — (DOCX) [file pmed.1003368.s002.docx]

**S1 Text** Example search strategy

Online search strategy for MEDLINE:

1) Complex systems:

“system* theory” OR “system* thinking” OR “system* science” OR “complex system*” OR “system* model?ing” OR “systems* dynamics” OR “system* approach” OR “system* lens” OR “system* perspective” OR complexity OR “complexity theory” OR “complexity science*” OR “complex adaptive system*” OR systems Theory/ OR systems Analysis/ OR nonlinear dynamics/

2) Evaluation:

Evaluat* OR “policy evaluat*” OR “prog* evaluat*” OR “formative evaluat*” OR “process evaluat*” OR “outcome evaluat*” OR context evaluat* OR evaluation studies as topic/ OR programme evaluation/

3) Health

"public health" OR "health promotion" OR "health inequality*" OR "health inequalities*" OR “health inequity” OR “health inequalities” OR "health behavio?r" OR "well-being" OR wellbeing OR nutrition OR obesity OR "fast food*" OR sugar OR salt OR tobacco OR smoking OR cigarette* OR alcohol OR "illegal drug*" OR "illicit drug*" OR "recreational drug*" OR "social determinant*" OR crime OR “community safety” OR transport* OR planning ADJ3 town OR planning ADJ3 city OR planning ADJ3 neighbo?rhood OR planning ADJ3 urban OR renewal ADJ3 city OR renewal ADJ3 neighbo?rhood OR renewal ADJ3 urban OR redevelopment ADJ3 town OR redevelopment ADJ3 city OR redevelopment ADJ3 neighbo?rhood OR redevelopment ADJ3 urban OR regeneration ADJ3 city OR regeneration ADJ3 neighbo?rhood OR regeneration ADJ3 urban OR revitali#ation ADJ3 city OR revitali#ation ADJ3 neighbo?rhood OR revitali#ation ADJ3 urban OR “urban health” OR housing ADJ3 improvement* OR home* ADJ3 improvement* OR rehousing OR “home* refurbishment” OR “housing modification*” OR “home modification*” OR “healthy home*” OR “healthy housing” OR “affordable housing” OR “affordable home*” OR “housing intervention*” OR education OR “whole school” OR school ADJ3 environment OR greenspace OR housing/ OR public housing/ OR crime/ OR city planning/ OR urban renewal/ OR education/ OR schools/ OR urban health/ OR fast foods/ OR tobacco/ OR smoking/ OR electronic cigarettes/ OR substance-related disorders/ OR street drugs/ OR alcohol drinking/ OR alcoholism/ OR “social determinants of health”/ OR public health/ OR health promotion/ OR health status disparities/ OR health behavior/ OR obesity/ OR smoking cessation/

Date range: 2014 – current (September 2019)

Limit: English language
