# Supplementary material for: Qualitative process evaluation from a complex systems perspective: A systematic review and framework for public health evaluators
Source: PLoS Med. 2020 Nov 2;17(11):e1003368. doi: 10.1371/journal.pmed.1003368 (PMC7605618; doi:10.1371/journal.pmed.1003368)
Supplement: S2 Text — (DOCX) [file pmed.1003368.s003.docx]

**S2 Text** Case studies

**Case study 1: Rothwell et al 2010 [41]**

**Aim:** To assess the implementation of the Welsh Network of Healthy School Schemes (WNHSS) at national, local, and school levels, using a systems approach drawing on the Ottawa Charter.

**Intervention:** The WNHSS is a health promotion intervention targeting pupils in Welsh primary and secondary schools. The Welsh Assembly Government gives funding to Health and Education partnerships in all Welsh local authorities, who in turn have Healthy School Coordinators (HSCs) who create and maintain local schemes. An Assembly Government official coordinates the scheme at a national level and has responsibility for the overall strategy, local accreditation, and training HSCs. Individual schools have in-school coordinators who work with the HSCs to carry out the activities considered a priority to the school and to progress through the different phases of the intervention.

**Design and methods:** The process evaluation adopted a cross-sectional case study design to assess the implementation of the program in its first six years. Data were collected at different levels within the system; a documentary review was conducted at the national level and semi-structured interviews were used to collect data at the local and school levels. A documentary review was conducted that included documents detailing national decisions on policy and funding for the program, consultation and policy documents, and Education Authority and Local Health Board policies. Evaluators also observed two national meetings for HSCs and interviewed the national coordinator of the WHNSS. To generate data at the local and school level, semi-structured interviews were conducted with HSCs from each of the local schemes and some participants provided evaluators with local or school-level documentation. Finally, three regional workshops were held wherein evaluators presented initial findings to participants from local education and health departments.

**Systems approach:** The evaluation adopted a “systems approach” and conceptualized the school-based network as a “complex adaptive system”. The evaluation team drew on a socio-ecological model to represent the system as comprised of three different *levels:* national, local, and school. The systems approach was evident in the evaluation design, sampling strategy, and analysis of findings. Specifically, the evaluation was designed to capture implementation processes at each of the three *levels* by collecting data from system *elements* at each level. Implicit in this framing was a *boundary* decision that bounded the system of inquiry to elements and behaviors within each of these three *levels.* The analysis focused on how the *relationships between elements* at the same level, and *interactions between elements at different system levels*, affected program implementation over time and between different sites. In doing so, the evaluators represented a range of different *perspectives,* although they describe in their limitations section that not all system element *perspectives* were represented; notably, no in-school coordinators were interviewed. Despite conceptualising the program and its context as a “complex adaptive system,” the evaluators only implicitly drew on one facet of complexity – *dynamism.* Implicit in the analysis was a *dynamic* nature of the intervention and the system, particularly as schools moved through different phases in the intervention. However, as the evaluators acknowledge, the evaluation was cross-sectional and therefore fairly static in nature, capturing a specific time point rather than collecting data at multiple points.

**Case study 2: Durie and Wyatt 2013 [42]**

**Aim:** To evaluate a learning program designed to create transformational community change, with a focus on the program’s implementation, the impact on the participants, and emergent community outcomes.

**Intervention:** Connecting Communities (C2) is asset-based community development approach that was developed by the Health Complexity Group. The aim of C2 was to create the context for service providers to consult with their communities and ensure that service provision adequately responded to community needs. The intervention involved 3 phases of inter-related components, which included case studies, workshops on practical skills and complexity theory, site visits, resident and service provider talks, and research workshops.

**Design and methods:**  The evaluation adopted a case study design that used several qualitative and participatory research methods. Semi-structured interviews were conducted with a range of actors in the system, including course designers, deliverers and participants. In addition, non-participant observation was conducted during course delivery, listening events, and community partnership meetings. Finally, participants were given opportunity to input into findings by clarifying and adding to summarized findings. The different methods were chosen to examine the dynamics of the system overtime as they changed and evolved in response to the intervention, as well as to focus on the relations between those living and working in the community.

**Complexity science:** The evaluators used complexity science as an underpinning theoretical framework for the intervention and evaluation, which extended from the evaluation design to data collection and interpretation. The local area was conceptualized as a “complex adaptive system” that has an open *boundary* so that the *boundary* between the community and the wider environment is considered fluid. The researchers focused their evaluation questions and analytical focus on the *“relations between the agents* [*elements*] in the system and their *interactions and relationships* with the system.” The evaluators designed the case study to collect data from a range of different participants in order to represent different *perspectives* in the system, operating within and across different system *levels.* The account of the program describes the *system trajectory* in which the evaluators depict how the *relationships and interactions* between system elements gave rise to *emergent outcomes,* including those that were *unexpected or unanticipated,* within the case study community*.* The evaluators trace *non-linear relationships* whereby actions and behaviors of system *elements* *feedback* and shape the intervention, its components, and the system itself. The analysis focuses on describing and analysing these examples of *adaptation* and *co-evolution*, with an emphasis on how the system’s *history*, *initial conditions* and *local rules* influence its *trajectory*. The analysis itself has a *dynamic* component, where the evaluators explore change in the community over time (a time period of two years).
