# Supplementary material for: Qualitative process evaluation from a complex systems perspective: A systematic review and framework for public health evaluators
Source: PLoS Med. 2020 Nov 2;17(11):e1003368. doi: 10.1371/journal.pmed.1003368 (PMC7605618; doi:10.1371/journal.pmed.1003368)
Supplement: Attachment — Submitted filename: mcgill.pdf [file pmed.1003368.s005.pdf]

This paper presents a systematic review of studies of process evaluation and complex systems.

I was asked for a statistical report and I interpret that to include all aspects of the design and conduct of the study. I understand that it has been reviewed before and I was asked to focus on the systematic review methods specifically. I have done this from scratch, I did not look at the previous reviews first.

Points of detail

Page 10 The authors have restricted themselves to studies published only in English. The message I draw from Grégoire et al. (1995); Moher et al. (1996); Egger et al. (1997); Jüni et al. (2002) is that bias is likely although it is hard to predict in advance the extent or direction of it. I would not necessarily commit the authors to translate everything but at least we should know what we are missing.

Page 10 Strictly speaking the figure called out here is part of the results.

Page 12 Again the table is results

Page 14 Most of the first paragraph on this page is also more results.

Page 14 When studies have given rise to duplicate publications how were they dealt with? Was all evidence collated from all of them or was one selected as the definitive one and if so how?

Points of more substance

The authors have restricted their search to 2014 and after and relied on two existing reviews for the period before that. This means that any evidence from the earlier time is that which was selected and verified by other people and not by the current authors. I feel that this cannot be optimal. There is even a view that one should not look at previous reviews at all because of the risks but I feel that is too strong.

Summary

Some comments of detail and one more important issue to address. After reading the previous reviews I have nothing to add to my initial review.

Michael Dewey

References

M Egger, T Zellweger-Zähner, M Schneider, C Junker, C Lengeler, and G Antes. Language bias in randomised controlled trials published in English and German. *Lancet*, 350:326–329, 1997.

G Grégoire, F Derderian, and J Le Lorier. Selecting the language of the publications included in a meta-analysis: is there a tower of Babel bias? *Journal of Clinical Epidemiology*, 48(1):159–163, 1995.

P Jüni, F Holenstein, J Sterne, C Bartlett, and M Egger. Direction and impact of language bias in meta–analyses of controlled trials: empirical study. *International Jounal of Epidemiology*, 31:115–123, 2002.

D Moher, P Fortin, A R Jadad, P Jüni, T Klassen, J Le Lorier, A Liberati, K Linde, and A Penna. Completeness of reporting of trials published in languages other than English: implications for conduct and reporting of systematic reviews. *Lancet*, 347:363–366, 1996.
