# Supplementary material for: Qualitative process evaluation from a complex systems perspective: A systematic review and framework for public health evaluators
Source: PLoS Med. 2020 Nov 2;17(11):e1003368. doi: 10.1371/journal.pmed.1003368 (PMC7605618; doi:10.1371/journal.pmed.1003368)
Supplement: Attachment — Submitted filename: Response to Reviewers.docx [file pmed.1003368.s004.docx]

**Editors’ comments:**

Requests from the editors:
1. Abstract – please include a sentence on the limitations of the study as the final sentence of the ‘Methods and Findings’ section.

***Response:*** We have added a sentence on the limitations of the study in the methods and findings section of the abstract. We also added a section to the end of the discussion in the manuscript itself to address these limitations in more depth (p.33-35).

2. Please avoid jargon (e.g., "with a complex system lens"; "relatively static descriptions of the system under inquiry") and explain more clearly what you found and what it might mean.
***Response:*** We have now simplified the abstract so that the jargon is removed and that it is clearer to a general audience exactly what we did, and the implications of this review work. We agree that the term ‘lens’ is quite jargon-y and have removed it from the manuscript entirely, replacing it with ‘perspective’.

3. Are you able to include the names of the "32 academics"?

***Response:*** We have written to the academics we contacted and have asked for their permission to be acknowledged for their contribution. At the time of submitting our revision, we had heard back from 7 and we have included their names in a new acknowledgement section at the end of the manuscript. This comment also prompted us to acknowledge the contributions to the search terms from the wider research team on the NIHR SPHR project out of which this review grew. Their names can also be found in the acknowledgement section.

4. At this stage, we ask that you include a short, non-technical Author Summary of your research to make findings accessible to a wide audience that includes both scientists and non-scientists. The Author Summary should immediately follow the Abstract in your revised manuscript. This text is subject to editorial change and should be distinct from the scientific abstract. Please see our author guidelines for more information: [https://journals.plos.org/plosmedicine/s/revising-your-manuscript#loc-author-summary](https://protect-eu.mimecast.com/s/EL3aCO8KBCqLxZU8FZ4I?domain=journals.plos.org)

***Response:*** We have now included an Author Summary directly after the abstract.

5. Please use sections and paragraphs instead of page numbers in the checklist – as these can change during revisions / formatting etc.

***Response:*** We have now used section and paragraphs in the PRISMA checklist.

6. You say in the PRISMA that the protocol is not in the public domain yes in the submission form in relation to data you say ‘This is a review of published studies; all studies are in the public domain. I would have thought if all studies are in the public domain the protocol would be too. Please explain.

***Response:*** We apologise for the confusion. All studies included in the review are published studies – so they are in the public domain. The protocol for our review was not published but we have now made it available online and provide the URL in the PRISMA table.

**Reviewers’ comments:**

***Response:*** Thank you to all the reviewers for reviewing our manuscript and for providing helpful and constructive comments. We have significantly revised the manuscript in light of these comments and believe they have strengthened the paper’s analysis, arguments, conclusion and presentation.

Reviewer #1:

1. I was asked to provide a statistical review of this paper. However, no statistics were used, so I have no comments on them
Peter Flom

***Response:*** Thank you for looking at the paper.


Reviewer #2:
This is a well-written, clear and very useful paper. It provides a nice conceptualization of complex systems thinking, and of static versus dynamic perspectives. The glossary and list of examples will be invaluable to persons working in this field.

1. My only substantive comment relates to the presentation of the 22 processes evaluations from the systematic review. These are well presented in the table and at a high-level in the main text and in Figure 2. They form a very useful repository of examples. However, short of digging out some of the references, I didn't come away with a clear picture of any exemplar of an evaluation that used systems thinking or complexity science. Perhaps due to word count it is not possible to present 1-2 examples in more detail: I leave that with the editor to consider.

***Response:*** We found this a very helpful suggestion and we have now written 2 ‘case study’ examples of evaluations. In line with comments from Reviewer #3, we decided to highlight one example of a study that applied concepts from systems thinking to produce a relatively static description of a system at a single time point. For our second case study, we chose an example that utilised a range of concepts from complexity science to explore the dynamics of a system undergoing change – in this study, complexity theory was used as a theoretical underpinning for both the intervention and the evaluation. We have currently included these two case studies as supplementary material given the length of the manuscript, but we would welcome a steer from the editors as to whether they would prefer us to include each case study as a box in the text or if we should leave them as supplementary files.

2. A few very minor comments.
Page 19, glossary, "coherence": "… functions aligns with other another". Please correct.
Page 18, "analytical framework" I believe should read "analytic framework".
Page 23, should read "data are collected…".
Page 26, Conclusions: Might the first sentence of this section better read "… informed by systems thinking and/OR complexity science.."?
Page 27, could you replace "evaluate" with another verb (eg assess) "we have therefor developed a framework for process evaluations that evaluate…"?
Page 27, might delete "ourselves", to read "we intend to test out this approach in the near future.".

***Response:*** Thank you for pointing all of these out. We have made all the suggested corrections.


Reviewer #3:

Thank you very much for providing me with the opportunity to review this interesting article on process evaluations with a complex system lens. It is the intention of the authors to develop a framework for researchers (?) who are planning the evaluation of a complex public health intervention.
While the scope of the manuscript is really interesting, I think the manuscript would benefit from some revisions.

Title:
1. Maybe you should integrate the focus on qualitative methods into the title, depending on how much you want to emphasize this component

***Response:*** We have integrated this into the title because this is a key aspect of the work.

Abstract
2. It does not become quite clear why complex interventions call for the application of qualitative methods; please formulate more precisely

***Response:*** We have briefly (because of the word count) addressed this in the abstract. In addition, we have made the argument more strongly in our background section (p.8-9) and introduced a new paper by Gear et al 2018 that speaks directly to the value to utilising complexity theory as a qualitative research methodology.

3. In the result section, you talk about analytical approach; not sure whether I would rather talk about conceptual approach or even underlying theory which is weaved into the entire process; is it systems concepts or system concepts? The last sentence of the result section is really hard to understand; can you be more precise?

***Response:*** We have revised this part of the abstract to consider both of these points. First, we have made it more explicit that theory and concepts from systems thinking and complexity science were used at different stages of evaluations (from design and planning through to the analysis). Second, we have substantially re-written the ‘Methods and Findings’ section – including the last sentence. We have also ensured that we used the term ‘systems concepts’ consistently throughout the manuscript.

- Conclusion:
4. Is it that methodological principles are underdeveloped? Or is it rather that methods (or even methodologies) do not justice to existing theories or conceptualizations of complexity? I think it is a great disconnect in public health literature with regards to the conceptualization of complexity and the methods (or methodologies) which are chosen to address those

***Response:*** Thank you for this helpful comment. We agree that the current application of qualitative methods does not currently do justice to existing theories and conceptualisations of complexity. We have amended the abstract text to take it into account. Specifically, we highlight the (to us) surprising finding that many studies described rather static, single time point, systems. We agree with the reviewer that this approach doesn’t really do justice to wider range of systems concepts. In fact, we think a ‘static system’ process evaluation is a rather counterintuitive approach for evaluators to take – given that both process evaluations and the study of complex systems should surely be geared towards examining how things change over time.

5. Concepts that should be operationalized: again, I think you need to be more specific

***Response:*** We have revised the text to be more specific. We agree that the word ‘operationalise’ can be unclear so we have removed it from the manuscript altogether.

6. I am not sure whether the focus should be on qualitative methods only; maybe it is more about integrating qualitative methods into the assessment of complex interventions alongside other methods. Maybe use the term "Integrate"

***Response:*** We believe the focus on qualitative methods is justifiable; many process evaluations are primarily qualitative in nature and even in instances where mixed methods are employed, there is still a need to understand and further develop what concepts and theory from systems thinking and complexity science can bring to the qualitative side of a mixed methods evaluation. Additionally, systems evaluations do not have to evaluate only complex interventions; indeed, even simple interventions may well produce varied and unpredictable impacts if introduced within a complex system. This is because a complex systems perspective takes complexity to be a property of the system rather than (necessarily) a property of the intervention implemented within that system. We agree with the reviewer’s point that we need to be clear that while our focus is on qualitative research that many of the evaluations we identified integrate qualitative methods into mixed methods process evaluations. We have made it clear in the manuscript that we are focusing on qualitative methods in process evaluations (see pages 7-9, 11), but that there are also examples that utilise mixed methods (although our focus remains on the qualitative methods). In addition, we have emphasised in our discussion section that our focus has been on qualitative research but that future research could expand more on the integration of qualitative and quantitative methods (p.33).

Background
7. I don't think you need the subheadings; I suggest rather to focus on connecting the concepts also in the background section

***Response:*** We have used three subheadings in our Background section and are concerned that dropping them would make the text harder to navigate – noting that the other reviewers were generally favourable towards the way the submitted draft was written. We believe the subheadings are useful for making the manuscript easy to read particularly as the Background section is relatively long. In line with the next comment (#8), we have done major revisions to the Background section to ensure the concepts are connected, while still maintaining the overarching structure. If published, we will ensure formatting (including subheadings) meets any further requirements from the editors.

8. Complexity: Maybe you can work on connecting the complex system and the complex intervention paragraph more closely; I would include the conceptualization of complex interventions as being considered events in complex systems, stress the interplay between the system and the intervention and that one can hardly be considered without the other.

***Response:*** We have now re-written large parts of the Background section with an emphasis on better connecting the concepts. We now introduce the concept of complex systems, before moving onto the roots within the systems thinking and complexity science traditions. We then use this to introduce the use of complexity within public health – distinguishing between public health’s use of the perspective to describe interventions or systems.

9. Evaluation: I think you need to specify other types of evaluations, such as outcome/impact evaluations; process evaluation is usually conducted alongside these evaluations; please provide rationales for each type of evaluation;

***Response:*** We have included this now on page 7-8 and explain that process evaluations are often conducted alongside impact/outcome evaluations.

10. Process evaluation definition: could you provide a definition of process evaluation? (e.g. Moore et al. 2015)

***Response:*** According to the MRC Guidance on Process Evaluations, there is no standard definition of a process evaluation. Moore and colleagues describe why process evaluations are undertaken and we have now included this in the manuscript (p.7), alongside our response to the previous comment.

Objectives:
11. Please be specific (also in the abstract) about what process evaluation approaches you are looking at: purely qualitative ones or the ones integrating qualitative approaches

***Response:*** Thank you for raising this. We agree our reporting may not have been entirely clear and we have now revised the text in several places to underscore that we included process evaluations that had a substantive qualitative component – therefore, both process evaluations utilising only qualitative methods were included, as well as mixed methods evaluations, as long as the qualitative component was substantive. We made these changes in the methods section of the abstract and we also have fleshed out the inclusion criteria (p.11) to ensure this is clear. The results section also highlights specifically which studies used purely qualitative methods and which integrated qualitative methods with quantitative ones – in all of these instances the qualitative results were substantive (as per the inclusion criteria) (p.20). Finally, we have amended Table 1 to include only the qualitative methods reported, so that this focus is clear.

12. The three goals are a bit misleading considering your objective; you were not looking for "types of public health interventions that have been subjected to process evaluations using a complex system lens" but the only the ones that have been assessed with (among others?) qualitative methods; be more precise about your objectives; also, the conceptualization or theory of complexity underlying the respective process evaluation approach was probably of interest to you.

***Response:*** We have revised our objectives in light of these comments (p.9). In our first objective we have specified that we were looking specifically only at those that had included a qualitative component. We revised our third objective in line with the suggestion here.

13. Be also more specific about the framework you aim to develop; you can refer to Nilsen et al (2015) for a terminology of framework in Implementation Science. Also add the intended audience to the objectives.

***Response:*** Thank you for the reference; we found the language and definitions in the Nilsen article helpful and have incorporated his terminology into the manuscript and we now reference the article. We have also added the intended audience for our framework (p.9).

Methods
14. Why did you choose Jan 2014 as starting point? Please provide rationale

***Response:*** We chose January 2014 because it captured evaluations that were published after the two systematic reviews that we hand searched; this rationale is included on page 10.

15. Add language limitation to the text

***Response:*** We have added that we only included English-language publications (p.10).

16. I am a bit surprised that your search strategy does not contain any search terms for qualitative methods; after having started reading the article in the abstract and the objectives, one would assume that this was a primary goal of the inquiry

***Response:*** We did not use a search filter to screen out non-qualitative methods because methodology filters carry their own risk of missing studies due to (over-)specificity. Not having the filter increases the sensitivity of the electronic search, giving the reviewers more scope to assess for themselves whether qualitative methods featured in the studies identified by that electronic search. In addition, we had a relatively broad search strategy because we had already conducted a wider scoping of all systems approaches to public health evaluation. It made sense to progress the current review by re-running that initial, sensitive search but adding an extended time period for publication (the time period we report in this review).

17. You should provide more details on your inclusion criteria; this is a conceptual review which means that including and excluding articles can become quite a challenge; it is therefore even more important that you provide us with your exact definition of each inclusion criteria, namely

***Response:*** Thank you for this overarching comment and the more specific details provided below. We respond below to each in turn, but we have approached this comment by expanding the section on our inclusion criteria (p.11). We also acknowledge that this process can be somewhat subjective; in many instances the decision to include or exclude was clear cut, but in other cases this required ongoing discussions between the researchers and multiple readings and re-readings of the evaluations in order to come to a decision. We have created a new limitations section in the Discussion section and we further consider this point there (p.33-34).

18. Self-identified system approach: where would that happen? In the background, method or discussion section? If you only use it to embed your findings, I don't assume this would be sufficient

***Response:*** We included studies that identified as using this approach at any stage in the evaluation process (planning and design, data collection, analysis, interpretation). This review scoped the literature on applying systems and complexity to process evaluations with qualitative methods and part of this process was getting an understanding about how, why and in what ways these traditions are drawn upon. We therefore did not wish to exclude evaluations that used these concepts as an analytical framework but who did not include this in the design stage (or did not report doing so). The reviewer’s comments around this point here and in other parts of the review have helped prompt us to be more explicit about one of the conclusions arising from our review – namely that many evaluations do not include systems and complexity concepts (or at least do not report doing so) from the design and planning stage. Our suggested evaluation framework is an attempt to prompt researchers to design these concepts into their evaluations from the beginning of the evaluative process, rather than just as a means to structure their analysis. We have brought the implications of this finding out more clearly in the manuscript.

19. Relevant to public health: I am not sure how to interpret that; was that a subjective judgement? How did you define relevance? How did you define public health? I could imagine quite some grey areas around this

***Response:*** We have now included the list of public health topic areas that we considered (p.11). However, as the reviewer points out, there are some grey areas so we did not exclude a study simply because the topic area did not appear on our list. If a reviewer felt it was a relevant public health area (most aspects of population health, excluding treatment), then a discussion was had with the other reviewer doing full text screening and also a third reviewer. We have made this process more explicit in the text. We have also acknowledged the limitations of this process in our discussion (p.33-34).

20. Process evaluations: did they have to be process evaluations only? What if it was an outcome evaluation alongside a process evaluation? Would that also be included? Again, also provide your definition and potential criteria you used

***Response:*** We have now expanded this section to clarify that process evaluations were included, as well as process evaluations alongside impact / outcome evaluations (p.11). In those instances, we focused only on the process evaluation component.

21. Qualitative methods: which definition did you use? I am wondering if methods such as document analysis etc. would be covered by your understanding of qualitative methods; also, would the process evaluation use ONLY qualitative methods, or would studies using mixed methods also be comprised? If so, please also provide your understanding of that (e.g. weighing of methods, sequence of methods etc. cp. Creswell et al.)

***Response:*** We considered a range of methods to be qualitative, including: interviews, group interviews or focus group discussions, (participant) observation, document review, free form responses on questionnaires and participatory and visual methods. We have added this list to the manuscript (p.11) and also make clear that we included evaluations that mixed methods components so long as the qualitative component was substantive – that is, so long as the evaluation generated and analysed qualitative data and reported those findings. In those cases, we included mixed methods studies, but only report on the qualitative component in this review.

- List of concepts of complexity science and system thinking:
22. it seems like this is where you lay out your understanding of complexity science and system thinking; you are considering them as two sides of one spectrum; I am wondering what spectrum this is and how "simplicity" fits in?

***Response:*** We are presenting the concepts as residing along a spectrum with systems thinking on the left-hand side and complexity science on the far right-hand side. This spectrum represents static to dynamic. With regards to simplicity, another way of looking at systems or interventions is a spectrum that moves from simple to complicated to complex and our understanding is that the forthcoming MRC Guidance on Developing and Evaluating Complex Interventions will work through this spectrum. We could have also worked with this spectrum, but we have rather chosen to focus specially on complexity and complex systems (rather than also include simple or complicated one) and our spectrum illustrates how one can describe, depict and analyse a system, moving from static to dynamics concepts and characteristics. We have added a sentence on page 14 to make this clear.

23. How was this list developed? Why were certain aspects chosen and others not?

***Response:*** We have now provided additional details about how we developed this list and the fact that there were subjective choices, although we aimed to represent the concepts most frequently described within each of the two traditions (p.12). We have acknowledged in our limitations section that a different research team may have made different decisions about what to highlight (p.33-34).

24. What is the difference between context, history and initial conditions? aren't local rules also context?

***Response:*** Thank you for this comment. We agree there is overlap between the terms and that the boundaries between them are not always clear cut – we have now stated this in the text. Based on this comment and further discussions amongst the research team, we have decided to remove the concept ‘context’ from the table. We have included a justification of this choice in the manuscript (p.12); we explain how arguably all features of a system are ‘contextual’ and as a result it is not a meaningful category in its own right. We also found that all the studies we identified had some consideration of context, which is hardly surprising given that all systems are contextual.

25. What is elements? How does it relate to context and levels?

***Response:*** We have defined elements as “Entities within a system, include, for example: people (‘agents’), organisations, resources, etc”. Depending on the system under inquiry and the evaluation approach, a system analysis might consider agents, variables or both and these agents (or variables) might operate at different levels within the system. A system will have both vertical and horizontal dimensions and an evaluator will make choices about which interactions between agents, and at which level(s), become the focus on the analysis.

26. Dynamics and interactions also seem to have some conceptual overlaps;

***Response:*** We agree with this point and we have added text about overlaps to both the description of generating the list (p. 12) and to the new limitations section of the manuscript (p.33-34). We note that while there is some overlap between ‘dynamics’ and ‘interactions’ that there are also features that distinguish them. Namely, interactions describe how system elements relate to each other and across system levels (or with elements outside the boundaries of the system of interest). These interactions can be described in a relatively static manner (i.e. described at a single time point), or rather the focus of the analysis might be on how the interactions evolve and change over time in response to system changes – here we are then considering dynamism as well, specifically focusing on the dynamic and evolving nature of interactions.

27. Summary: Did you take a look at other conceptualizations of complexity, such as [https://www.ncbi.nlm.nih.gov/pubmed/28446138](https://protect-eu.mimecast.com/s/fH5LCPQLDUy1XNCgi-y2?domain=ncbi.nlm.nih.gov) ? It seems that there is quite an overlap between some of these concepts; it is hard to understand the rationale for some of these concepts and their implication for evaluation

***Response:*** Thank you for the reference. This was not one of the papers that we included in our list to generate the concepts because it deals specifically with complex interventions, rather than complex systems. As we have argued previously, we are interested in the complex nature of the system and believe that both simple and complex interventions may generate complex responses within the systems into which they are introduced. We agree that there is overlap between many concepts and we have acknowledged that now in the manuscript explicitly.

28. I would call the section on quality appraisal something like critical appraisal. Could you be more specific about the ratings? At what stage would any of these concepts be applied? Would it be important to know whether a "complexity lens" was applied at the conceptualization or design stage? Would this make a difference compared to applying it at the analysis/interpretation stage when all data has already been collected?

***Response:*** In line with this recommendation, we have now created a sub-section called ‘critical appraisal.’ We agree that this is not a quality appraisal and we think that the term ‘critical appraisal’ applies to how we analysed the data. For the process of critical appraisal, we did not distinguish between when within the planning/design and conduct of the evaluation that the systems or complexity lens was applied. We have now made that clear in the text (p.13).

29. The understanding of "application" of a "complexity lens" seems quite fuzzy to me; Can you be more specific about both terms?

***Response:*** We have expanded this section by 1) being clear that this could happen at any stage in the evaluation (planning/design, data collection, analysis, interpretation) and 2) providing an example of what would earn a green code (p.13)

30. Throughout the analysis/synthesis section, I am not quite sure how the qualitative methods aspects comes in; how were the two concepts - complexity and QRM - - connected?

***Response:*** The review considers how concepts from systems thinking and complexity science have been used as theoretical underpinnings in the course of qualitative process evaluative research. In this sense, they are connected because the theoretical underpinnings should guide the way in which the evaluation is conducted, including how and from whom data are collected and the ways in which findings are analysed interpreted and presented. On page 20 we give examples of how the identified evaluations used complex systems thinking to guide their choice and application of certain methods. We have expanded this section in our revised manuscript to draw this out even further. In addition, we used the new case studies (suggested by Reviewer #2) to give more details on the qualitative methods used in two studies and how they were informed by a complex systems lens. Finally, in order to bring a sharper focus to the issue of qualitative methods in the revised manuscript, in the background section, we now have an additional paragraph that is designed to introduce some of these qualitative issues to the reader.

31. What I think is missing is the actual method extraction: what was the mode of evaluation, which time points, which methods at which time point, at what stage complexity lenses have been applied and so on; this could also be integrated into the result section; just think about your audience: what would a person who wants to evaluate complex interventions want to know?

***Response:*** We have addressed this comment in four ways. First, in order to address ‘mode of evaluation’ we have added a paragraph in the results section about whether or not these process evaluations accompanied impact evaluations (p.20-21). Second, we found that the papers were often not very clear about when methods were employed (and most were conducted at one time point, often poorly specified). We have added a recommendation in the discussion section that evaluators need to consider and report the timing of their choice of methods (p.31). Third, we have expanded a column in Table 1 ‘Complex systems perspective and evaluation stage’ to say exactly where a complex systems perspective was applied within the evaluation (ranging from design to planning). Fourth, we have also added a paragraph to the results section that summarises our findings in this area (p.19).

Results
32. What is public health strategy? It does seem to fall out of line with the other fields of application

***Response:*** We agree the language we originally used was confusing. We have now been more specific about the type of public health interventions were describing – these changes are in the results section (p.14) and Table 1.

33. Disciplinary background? I assumed the disciplinary background was public health? Or are you referring to the primary authors?

***Response:*** We understand that this is confusing and we have removed this from the manuscript. We have removed it from the results, as well as from Table 1.

34. Studies drew from… - with regards to what?

***Response:*** In line with the previous comment, we have removed this sentence from the manuscript.

35. Can you provide an overview of all complexity theories applied?

***Response:*** The review provides an overview of all the complexity concepts used in the analysis. We can do that for every included study. We cannot provide an overview of all the complexity theories applied to each study because many of the studies did not specify this. They may not have reported the theories they used although we suspect that many simply developed their own ad hoc approach rather than drawing substantially on an existing framework. When studies did specifically report that they were drawing on a particular theory we have specified this in the Complex systems perspective and evaluation stage column of table 1. For example, it includes “Cynefin” or “Dynamics systems framework” and others.

36. Can you provide an overview over the stages at which the frameworks or theories have been applied?

***Response:*** We have now provided an overview of this in Table 1 by providing more detail in the column ‘Complex systems perspective and evaluation stage’.’ The text is now clear about at what stage the any systems or complexity-associated framework or theory was applied (i.e. in the design, sampling strategy, design of data collection tools, analysis and/or interpretation of findings) (p.19).

37. Were the frameworks you report only used for structuring the analysis or already at the planning stage?

***Response:*** We found that this was mixed within the studies. Many studies did begin drawing on systems thinking and complexity (through, for example envisaging the system and its components, and focusing specifically on relationships) at the evaluation planning stage. In some cases, this was represented in the sampling strategy (e.g. conscious effort to sample different elements or from different levels within the system). Some studies drew on systems and/or complexity concepts when designing their data collection tools (e.g. interview topic guides). A number of studies used (complex) systems concepts or frameworks solely to structure the analysis. An overview of this is now provided in Table 1 in the column ‘Complex systems perspective and evaluation stage’. We have also added a new paragraph to the results section which summarises our findings in this area (p.19). We also argue that being explicit about how and when systems thinking and complexity science informs evaluation planning is needed. This was not a conclusion that we had drawn out before, but we think that it is an important one, so thank you for your comment that has prompted this. We have added this conclusion to the manuscript.

38. From a researcher' point of view, I think it would be interesting for what purpose one or more of the qualitative methods have been employed; what was their sequence? How did they inform other components of the evaluation? E.g. document analysis becomes more and more comment, but is used for different purposes; at what stages were they used (baseline, interim, final)? Did the purpose differ?

***Response:*** Thank you for this comment which has prompted us to reflect more on this point. We found that the studies often did not give clear rationales for their choice of methods, and particularly for the rationale for using a mixture of different methods (p.27). We have added some suggestions for how researchers can consider the value of mixing different qualitative methods (p.31).

Discussion
39. Moving from a review to guidance is quite a normative process; I am not entirely sure whether I would put this into the discussion or the result section; if put into the result section, it should be made clear how this framework came about; as an alternative, the framework could represent a summary of current practice, with each of the steps being informed by included studies; you could use this framework as a starting point and check which of the included studies followed this process. Maybe there is a step that you have missed? You could then discuss potential missing steps/aspects in the discussion

***Response:*** The suggested framework is a kind of recommendation and recommendations are usually found in the Discussion section. We think the framework should remain in that section. We agree that the pathway from review findings to the rational underpinning the framework needed to be more clearly specified. We have completely re-written to first part of the Discussion to do this. Here is the key paragraph which outlines our critique of approaches taken by the studies we reviewed.

From this summary of the reviews main findings we suggest that approaches to designing, conducting and reporting qualitative process evaluation that have complex system perspective are frequently under-developed and poorly specified. It is unclear to what extent systems thinking and complexity science influenced the key evaluation stages of study design, sampling and data collection. The underlying theories informing evaluations are often unclear. The tendency to focus on system concepts that describe a static system, rather than those best suited for assessing system change seems counter-intuitive – given that process evaluations are intended to assess mechanisms of change. We note that this rather critical assessment applies to many but not all of the studies we identified.

We then go on to explain how the two-stage framework we have developed is designed to address the methodological weaknesses identified from the review.

40. Use of theory of change or logic model should be strongly encouraged

***Response:*** We have now emphasised this point in our Phase 1 description (p. 28).

41. Qualitative research usually has a strong theoretical underpinning; how does complexity theory align with other theoretical perspectives (e.g. poststructuralism)? How does complexity theory augment qualitative research methods?

***Response:*** We have now discussed in more detail in our discussion how choosing a complexity-consistent theoretical underpinning can enhance the application of mixed qualitative methods (p.31). We believe that complexity theory is compatible with a range of methods, including quantitative and qualitative methods. Rather than the theory augmenting qualitative methods, the use of qualitative methods augments our ability to understand different dimensions and complex behaviours. Complexity theory is one of many theoretical perspectives, while there are areas of overlap with other theoretical perspectives, the uniqueness can be found in its conceptualisation of reality as best described as a system of interconnected elements that have certain properties, boundaries and behavioural patterns. While some of those characteristics or behavioural patterns (e.g. emergence etc.) can be found in other theoretical approaches, this theory has systems and complex adaptive behaviour at its core. Currently there are philosophical and conceptual debates surrounding the placement of complexity science within other paradigms (e.g. postmodernism and post positivism).  For example, there is some who argue that complexity science is best placed within the realist ontology, however it is also an area of vigorous debate and outside the scope of the paper.

42. I wonder how the respective "complexity lens" and the chosen qualitative research methods relate to each other? Were they chosen based on the lens or was it rather a purposive choice?

***Response:*** We found that not authors articulated why specific methods were chosen, although there was some rationale given in some of the papers. We have now reported this as a finding in the results section (p.20) and also added in the discussion that there should be both a clear rationale and clear reporting of these decisions (p.31).

43. In order to claim a complexity perspective, I think all of the evaluations need to have a complexity perspective from the very beginning; I didn't go through the primary studies, but maybe this is something worth taking a second look at

***Response:*** All of the studies have introduction sections that do delve into systems and/or complexity. What is unclear, however, is the extent to which this then informed the entire evaluation design, or was included because an evaluation subsequently used systems thinking to inform the analysis (even if this was not planned at the evaluation design stage). Part of this is an issue with reporting – something we now flag in our results section (p.19). We found that many evaluators however, do at least implicitly (although also often explicitly) integrate a complex systems perspective from the beginning of their evaluation design and this is evident in their choice of research question, sampling strategy and/or design of data collection tools. Other evaluations we identified did not make this clear or were clear that they only used a complex systems framework or perspective within their analysis of their findings. However, we do not think that this latter type of evaluation should be excluded. The aim of our review is to present the base of literature to date, analyse it and suggest an evaluation framework to progress the field. As such, we are aiming on the side of inclusion to give a clear representation of the literature base at this stage. In line with the comment here, and some above, we now make explicit in our results section (p.19) and Table 1 in which stages of evaluation the complex systems lens is applied. And finally, in our discussion (p.27) and conclusion sections we argue that such a perspective should be used from the very beginning of any evaluation (and clearly reported) (p.35).

44. What you should also take another look at is the overall development, implementation and evaluation of a complex PH intervention process; do qualitative methods only come in at the evaluation stage? When does evaluation start?

***Response:*** Our focus was on evaluation, but some of our studies did provide some data on intervention development, and there are instances where a complex systems lens influenced the intervention design, or instances of a developmental evaluation approach where findings from the evaluation were intended to inform further intervention development – in these instances the qualitative methods informed the development and implementation of the intervention. We have reported on this in Table 1 in the column now labelled ‘Complex systems perspective and evaluation stage’ – for example, Burman & Aphane use the complexity-informed analysis for further intervention development. We also found instances where a complex systems lens was evident from the intervention development stage and this followed through to the evaluation design and conduct. On the basis of the comment here and the recommendation from Reviewer 2, we have now written two case studies. One of our case studies (Durie and Wyatt) was chosen as an example of when complexity theory was used for both the intervention development and the evaluation.

Table 1:
45. Please specify roots; and also be consistent with the text

***Response:*** In line with a previous comment (#33), we have removed this from the manuscript.

46. Application of complex systems: What does that mean? Please be more specific

***Response:*** We have re-named this column to be more specific. It is now called ‘Complex systems perspective and evaluation stage’. In addition, we have provided clarifying text in the methods section (p.12) to make clear that the aim of this column is to show 1) what aspect of systems and/or complexity thinking features in each evaluation) and 2) in what stage of the evaluation it featured. This change in focus was in response to previous comments (#36, 37, 44) regarding the stages of evaluation.

47. System map: what is this? Please also put into text!

***Response:*** We have now written out a system map description in the methods section (p.12) so it is clear what we are describing. This is also where we now denote whether the evaluation had a logic model. We have now included a paragraph in the results section that describes the system maps (p.21).

48. Could you describe the types of evaluative approaches that have been undertaken unless all of them are process evaluations only? Were they all continuous? How many points of measurements did they have? And did each of the measurement points comprise the same methods?

***Response:*** We have now added a paragraph into the results section where we describe whether authors report an associated impact evaluation (p.21).

49. I would also put in the stage at which a complexity lens was used

***Response:*** We have added this in column ‘complex systems perspective and evaluation stage’ in Table 1.

50. Maybe also add whether or not a logic model was used (quite standard in complex interventions)

***Response:*** We have now included if the authors presented a logic model or depiction of their programme theory. This is in the column labelled ‘map’ in Table 1.

Flow Chart:
51. you excluded studies that were not primarily qualitative, but then the result sections reports mixed method studies? How does that fit together?

***Response:*** We have now amended the flow chart (Fig 1) to say studies that ‘were not substantively qualitative’. The rationale for this is above and in the revised manuscript (briefly, we included mixed methods evaluations as long as there was a substantive qualitative component and we focussed only on the qualitative findings in this review) (p.11).

52. Not public health? How does that fit with the disciplinary background?

***Response:*** We have re-written this to say “no public health relevance” to make clear that we were interested only in evaluations of interventions with a clear public health relevance. Based on comment #19, we have expanded the inclusion criteria section to outline exactly what we meant by ‘relevant to public health’ (p.11).

53. What does "not empirical finding" mean? Why has that not been included in the inclusion criteria?

***Response:*** Thank you for pointing out that we had not included this in our inclusion criteria. This was one of our inclusion criteria and we have now included it in the methods section (p.11).

54. Figure 2: you could consider using the Cochrane RoB display as alternative to this graphic display; it is a bit easier to understand

***Response:*** Thank you for this suggestion. We have now re-formatted the figure (Fig 2) in a manner that is similar to the Cochrane RoB display and we hope that this now makes it easier to understand and read. We have not exactly replicated the Cochrane RoB format, however, because our tool is a form of critical appraisal, rather than risk of bias assessment tool and we do not wish to confuse the two.

Reviewer #4:

This is a very interesting and well written article, which I think makes an important contribution to the methodological literature around process evaluation and systems thinking.

I have a two quite minor and general comments regarding some of it's framing, limitations and contribution to the literature which the authors could consider.

1. First, it would be good to see some more extended reflection in the discussion about the limitations of focusing on studies which self-identify as using systems perspectives. Because the focus is limited to qualitative studies, systems thinking is likely to be more prevalent than complexity science. However, as the authors set out, this is a way of thinking and seeing the world more than a discipline or method. Hence, it is perfectly possible for authors in deciding their research questions and planning their studies to be guided by ways of thinking which are compatible with systems thinking, without ever using that term and citing the systems literature. In fact, many elements of systems thinking can probably be retrospectively identified within most good quality qualitative research. My sense is that what the field is trying to do at the moment, which in itself is a major attempt to disrupt the evidence production system, is to normalise systems thinking in evaluative
practice such that it no longer becomes a separate bounded approach to doing evaluation and just becomes what people do. For many in this field, that is perhaps already the case. On the other hand, during this period of system disruption, lots of people began to use the term systems thinking in the past 5-10 years without really engaging with its core concepts, just because it has become something of a fashionable buzz-phrase and they thought they needed to say they were doing it to get funded and published. So I think a useful next phase to take this work further would be to identify and compare qualitative or mixed method process evaluations which do or do not explicitly cite adopting a systems framework, and delineate what value if any is added by that more explicit engagement with the systems literature, and how much systems thinking compatibility is emerging in studies which don't assign that label to themselves explicitly.

***Response:*** Thank you for this comment. We agree with the points raised here and find the suggestions for future research particularly helpful. We have now added a limitations section (p.34-35) which outlines what we see are the two main critiques here: first, that a focus on self-identified complex systems studies misses out research that is compatible with systems thinking (particularly a lot of rigorous qualitative research), and second, that as a focus on complex systems has become more fashionable, that researchers may apply the terminology in an uncritical manner.

2. I think there could be a little more recognition that many of the issues being discussed here were present within 2014 MRC guidance for process evaluation, which did pay attention to the need for flexibility to respond to emerging issues, and explicitly put context front and centre as the largest box within the diagram, with pre-existing contextual/system conditions framing everything that followed in terms of what kinds of intervention were selected, what mechanisms they activated and what outcomes occurred as a consequence. It maybe shied away from bringing a complex systems perspective too much to the forefront due to a desire to take people along with it, rather than leaving people behind by being too radical. But I understand the forthcoming MRC guidance which we should expect to see sometime in 2020 will be more bold in that regard, reflecting how the evidence production system has shifted over time. More recently, this article has revisited some of the systems-consistent elements of this guidance (Moore, Graham, Evans, Rhiannon, Hawkins, Jemma, Littlecott, Hannah, Melendez-Torres, Gerardo, Bonell, Chris and Murphy, Simon 2019. From complex social interventions to interventions in complex social systems: future directions and unresolved questions for intervention development and evaluation. Evaluation 25 (1) , pp. 23-45. [https://journals.sagepub.com/doi/10.1177/1356389018803219](https://protect-eu.mimecast.com/s/LiwMCQ7METz1PlhL9Bpf?domain=journals.sagepub.com)) and actually I think there is a lot of agreement between the recommendations of your review and new framework, and some of the statements within that article around the need to understand interventions explicitly as attempts to change how systems function, starting by understanding the system before focusing on how a new way of working alters it and what consequences that produces etc...

Graham Moore

***Response:*** We agree with this and we thank the reviewer for the paper, which we should have included in the original manuscript. We have now cited the paper several times in the revised manuscript and we agree that many of the issues raised in that paper are in agreement with our own arguments. This includes, for example that there have been few attempts to show what a complex systems approach would look like in practice (p.27), that evaluators should critically consider which complexity concepts will help produce better evidence, rather than trying to somehow apply them all (p.30) and that evaluations applying a complex systems perspective may require longer time scales in order to capture complex system dynamics in response to an intervention. We now say explicitly in our discussion section that the MRC Guidance is compatible with systems thinking (p.34), particularly with the emphasis on foregrounding context and understanding the dynamic interplay of relations between system actors.
