# Supplementary material for: Qualitative process evaluation from a complex systems perspective: A systematic review and framework for public health evaluators
Source: PLoS Med. 2020 Nov 2;17(11):e1003368. doi: 10.1371/journal.pmed.1003368 (PMC7605618; doi:10.1371/journal.pmed.1003368)
Supplement: Attachment — Submitted filename: Response to Reviewers.docx [file pmed.1003368.s006.docx]

**Editors’ comments:**

***Reponses:*** Thank you for the opportunity to revise and further strengthen our manuscript. We appreciate the time you and the reviewers have put into reviewing the paper and we are pleased to submit our revision.

1. Abstract Background: perhaps there is an alternative to using “review” twice in the same sentence.

***Response:*** We have changed the second ‘review’ to ‘analyse’.

2. Please ensure that all references use the "Vancouver" style for formatting, and see our website for other reference guidelines [https://journals.plos.org/plosmedicine/s/submission-guidelines#loc-references](https://protect-eu.mimecast.com/s/0IoeCO8KBCpZyAoFp2ARN?domain=journals.plos.org)

***Response:*** We have gone through the reference list carefully and made any changes needed to conform to the Vancouver style of referencing.

3. Please remove the contraction from the 1st point of the Author Summary.

***Response:*** We have removed the contraction.

4. Please add “to our knowledge” or similar to the 4th point of the Author Summary.

***Response:*** We have added ‘to our knowledge’ to this point.

5. Author Summary, 7th point: please be more specific than “a lot”.

***Response:*** We have been more specific by using the phrase ‘the majority’ which mirrors the language used in the manuscript.

6. Please rename the “Background” section “Introduction”.

***Response:*** We have renamed this section as ‘Introduction’.

7. Please describe how the 32 academics were identified.

***Response:*** We identified academics who are active in the application of complex systems approaches to public health – including those in our own professional networks as well as those whose work we have read. We then set out consultation emails to those we identified. We now give more detail on this on page 10. Since our previous revision, an additional academic (Laurence Moore) whom we consulted has been in touch to be included in the Acknowledgements section and we have added his name there.

**Reviewers’ comments:**

***Response:*** Thank you to all the reviewers for taking the time to review our revised manuscript and provide constructive suggestions on how to further improve the paper.

Reviewer #3:

Thank you for providing me with the opportunity to review this manuscript once more - after having been particularly picky around the methods. My apologies, this only happens when I find things particularly interesting and really think about what implications this has on our research practice.

I went through the comments provided by other reviewers and re-read the manuscript. I think the manuscript improved very much and I would like to acknowledge the efforts that went into the revision of the manuscript. In particular I enjoyed reading the authors reflections surrounding the role of complexity theory in paradigm discussions in QRM.

***Response:*** Thank you again for your comments on the original manuscript and these additional comments below. We have found your comments insightful and challenging and addressing them has improved the manuscript.

I have three minor comments:
1. I appreciate the efforts that went into revising the methods section; by specifying your inclusion criteria and acknowledging the many grey areas around the concepts which determined inclusion or exclusion, I think you already contribute to moving the discussion forward; I would still encourage the authors to link their conceptual work to existing conceptual work around public health, qualitative research methods and mixed methods (e.g. substantial component is quite subjective; there is however literature which describes the role of qualitative research in mixed method which you could use as a basis to further specify your inclusion criteria. If you feel that this is too much text, you could also do this in tabular form.

***Response:*** We have now further expanded our inclusion criteria in order to address this point (p.11-12). Specifically, we have referenced Cresswell’s work on mixed methods design and the MRC Guidance on Process Evaluations section on mixed methods. We used these to elaborate on the different types of mixed methods designs that were eligible for inclusion and we have further defined what we mean by ‘substantive component’. Based on this comment and the reflection it prompted within the research team, we re-applied the criteria to all of our included studies and ultimately decided to remove the Igras et al (2014) paper from the review as it does not meet the criteria to be ‘substantively qualitative’. This has been reflected in the manuscript and accompanying figures.

2. Page 13: maybe use "yellow" rather than "amber", cause it might be easier to understand.

***Response:*** We have made this change and we have also amended the figure to change the colour from an amber/orange to yellow.

3. "The aim of this analysis was not to be overly critical about individual studies, but rather to understand the ways in which concepts from systems thinking and complexity science are applied in this body of literature" --> maybe substitute analysis by appraisal

***Response:*** We have changed ‘appraisal’ to ‘analysis’ on page 14.

I am looking forward to seeing this published and taken up by the research community.


Reviewer #4:

I very much enjoyed reading this paper, and I think it represents a very important and timely methodological contribution
***Response:*** Thank you very much for reviewing the paper and for your comments.

Reviewer #5:

This paper presents a systematic review of studies of process evaluation and complex systems. I was asked for a statistical report and I interpret that to include all aspects of the design and conduct of the study. I understand that it has been reviewed before and I was asked to focus on the systematic review methods specially. I have done this from scratch, I did not look at the previous reviews first.

***Response:*** Thank you for reviewing the methods of our paper; we address your specific points below and we believe that incorporating your comments has improved the manuscript.

Points of detail:

1. Page 10 The authors have restricted themselves to studies published only in English. The message I draw from Grégoire et al. (1995); Moher et al. (1996); Egger et al. (1997); Jüni et al. (2002) is that bias is likely although it is hard to predict in advance the extent or direction of it. I would not necessarily commit the authors to translate everything but at least we should know what we are missing.

***Response:*** Thank you for the references and for calling attention to this limitation. We agree that it is possible the inclusion of only English-language publications in a conceptual and methodological review like ours is a limitation. Articulating this in a review like ours is particularly tricky. If this were an effectiveness review (which, of course, it is not), a great deal of concern would rightly be focused on the precision of the synthesised quantitative estimate(s) of effect, and the potential for bias to push the estimate towards a false conclusion about intervention effectiveness. In our review, it seems to us that the main limitation lies in potentially missing studies that might shed further light on methodological approaches, which could further inform our methodological discussion and recommendations. We have referred to this limitation in our abstract (p.2) as well as the discussion section (p.37). We note that the review has not recommend we include these studies now, but rather make an explicit statement about the limitation.

2. Page 10 Strictly speaking the figure called out here is part of the results.

***Response:*** We have removed reference to the figure from this part of the methods. The first time the figure is now referenced is in the results section on page 14.

Page 12 Again the table is results

***Response:*** We referenced the table within the methods section as a way of signposting the reader to the table and its contents, but the actual table does not appear until the results section (p.26-27).

Page 14 Most of the first paragraph on this page is also more results.

***Response:*** We have moved this paragraph to the results section where we introduce and show the figure (p.22).

Page 14 When studies have given rise to duplicate publications how were they dealt with? Was all evidence collated from all of them or was one selected as the definitive one and if so how?

***Response:*** When studies gave rise to duplicate publications, we read and extracted data from across all the identified studies. We have now explained this process on page 12.

Points of more substance:

The authors have restricted their search to 2014 and after and relied on two existing reviews for the period before that. This means that any evidence from the earlier time is that which was selected and verified by other people and not by the current authors. I feel that this cannot be optimal. There is even a view that one should not look at previous reviews at all because of the risks but I feel that is too strong.

***Response:*** Thank you for raising this point for our consideration. We believe that this point is most applicable to systematic reviews and meta-analyses concerned with effectiveness (or other quantitative reviews – e.g. of risk factors), where there is a risk of bias to the precision and accuracy of the effect size estimate. It is harder to know what bias could be introduced by our methods for a review of concepts and methods, such as the one we have undertaken. We agree that in theory this could affect our judgements, but this would unlikely have the same impact as with a review of effectiveness (see our response to your first point, above). We also note that the application of complex systems thinking within public health is something that has really taken off over the past several years and therefore the studies that meet our inclusion criteria are more likely to be recently published and therefore captured in our systematic search. We also did not rely solely on the previous two reviews, but also consulted widely with academic experts (n=32) active in this field who provided us references to studies or work they deemed relevant. Despite this, we do agree that there is the possibility of us having missed earlier studies due to how we structured our search and this is an important limitation to acknowledge. We have now done so clearly in our revised manuscript (p.37).

Summary:

Some comments of detail and one more important issue to address. After reading the previous reviews I have nothing to add to my initial review. Michael Dewey

***Response:*** Thank you again for your review and helpful comments.

References

M Egger, T Zellweger-Zähner, M Schneider, C Junker, C Lengeler, and G Antes. Language bias in randomised controlled trials published in English and German. Lancet, 350:326-329, 1997.

G Grégoire, F Derderian, and J Le Lorier. Selecting the language of the publications included in a meta-analysis: is there a tower of Babel bias? Journal of Clinical Epidemiology, 48(1):159-163, 1995.

P Jüni, F Holenstein, J Sterne, C Bartlett, and M Egger. Direction and impact of language bias in meta-analyses of controlled trials: empirical study. International Jounal of Epidemiology, 31:115{123, 2002.

D Moher, P Fortin, A R Jadad, P Jüni, T Klassen, J Le Lorier, A Liberati, K Linde, and A Penna. Completeness of reporting of trials published in languages other than English: implications for conduct and reporting of systematic reviews. Lancet, 347:363{366, 1996.
