# Supplementary material for: Qualitative process evaluation from a complex systems perspective: A systematic review and framework for public health evaluators
Source: PLoS Med. 2020 Nov 2;17(11):e1003368. doi: 10.1371/journal.pmed.1003368 (PMC7605618; doi:10.1371/journal.pmed.1003368)
Supplement: Attachment — Submitted filename: Response to Editors and Production Team.docx [file pmed.1003368.s007.docx]

Dear Editors and Production Team,

Thank you for your additional editorial and production requests. We have made the changes requested and give point-by-point responses below.

**EDITORIAL REQUESTS:**

1. Thank you for editing the start of the Abstract Conclusions. However, please re-cast this section to more explicitly state the current study’s findings, followed by the implications.

Suggestion:

“This study found no consensus on what bringing a complex systems perspective to public health process evaluations with qualitative methods looks like in practice, and that many studies describe static systems at a single time point. We suggest future studies utilize a two-phase framework for qualitative process evaluations that seek to assess changes over time from a complex systems perspective. The first phase involves producing a description of the system and identifying hypotheses about how the system may change in response to the intervention. The second phase involves following the pathway of emergent findings in an adaptive evaluation approach.”

***Response:*** Thank you for this steer; we have made the suggested changes to the paragraph, with a minor tweak in the wording. The paragraph now reads:

“This study found no consensus on what bringing a complex systems perspective to public health process evaluations with qualitative methods looks like in practice, and that many studies of this nature describe static systems at a single time point. We suggest future studies use a two-phase framework for qualitative process evaluations that seek to assess changes over time from a complex systems perspective. The first phase involves producing a description of the system and identifying hypotheses about how the system may change in response to the intervention. The second phase involves following the pathway of emergent findings in an adaptive evaluation approach.”

2. Thank you for including the names of the academics who agreed to be acknowledged. Please note in the Methods section that the original consultation did not request permission to be named, but that those who did provide permission during the review process are named in the Acknowledgments. In the Acknowledgments, please also include the institutions for the academics named.

***Response:*** We have now added a note in our Methods section that reads: “In the original consultation we did not request permission to be named, but those who did provide permission during the review process are named in the Acknowledgements.” In addition, we have added the academics’ institutional affiliations.

**SUPPORTING INFORMATION:**

1. Please ensure that all Supporting Information files are clearly labeled in the file inventory. i.e. S1_Fig.tif, S1_Table.xlsx, S1_Text.doc, S1_Data.xlsx, S1_PRISMA_Checklist.docx, etc.

Each file type should be numbered starting at 1. Please also make sure that the file description matches the file name (e.g. S1_Fig.tif is listed as S1 Fig).

***Response:*** We have made these changes; specifically, now each different file type starts at 1 (so we have an S1_Text, S1_PRISMA_Checklist, and S2_Text as file names.

2. Please note that must refer to specific files using our naming convention (S1 Fig [not S1 Figure], S1 Table, S1 Text, S1 Data, etc.) Please update all in-text citations and supporting information file legends/captions accordingly.

***Response:*** We have used the naming convention and updated the in-text citations accordingly.
